# GPs’ experiences of phosphatidylethanol in treatment of hypertension: a qualitative study

**DOI:** 10.3399/BJGPO.2023.0037

**Published:** 2023-11-15

**Authors:** Åsa Thurfjell, Christina Sandlund, Johanna Adami, Jan Hasselström, Maria Hagströmer, Lena Lundh

**Affiliations:** 1 Division of Family Medicine and Primary Care, Department of Neurobiology, Care Sciences and Society, Karolinska Institutet, Solna, Sweden; 2 Academic Primary Health Care Centre, Region Stockholm, Stockholm, Sweden; 3 Division of Physiotherapy, Department of Neurobiology, Care Sciences and Society, Karolinska Institutet, Solna, Sweden; 4 Sophiahemmet University, Stockholm, Sweden

**Keywords:** alcohol drinking, glycerophospholipids, hypertension, general practitioners, primary health care, qualitative study

## Abstract

**Background:**

Hazardous alcohol use increases the risk of hypertension but is underdetected in primary healthcare (PHC) patients. Use of the biomarker phosphatidylethanol (PEth), which reflects the last 2–3 weeks of alcohol consumption, is increasing in Swedish PHC, but studies exploring its use for hypertension are scarce or missing.

**Aim:**

To explore GPs’ experiences of using PEth to identify hazardous alcohol use in the context of managing hypertension.

**Design & setting:**

A qualitative study of GPs (*n* = 12) experienced in using PEth in hypertension management who were recruited at Swedish primary healthcare centres (PHCC) in 2021.

**Method:**

The GPs participated in five focus group interviews. A questioning route was used. The interviews were audio-recorded, transcribed verbatim, and analysed with inductive qualitative content analysis.

**Results:**

*'I don’t hesitate anymore'* was the overall theme, which reflected both the disappearance of GPs’ fear that the PEth result might upset the patient, as this rarely occurred, and that the positive effects of PEth predominated in the findings. The theme is underpinned by the following four sub-themes: serving as an eye-opener; improving the dialogue; using with care; and learning by doing.

**Conclusion:**

PEth is a useful tool that changed GPs’ routines for addressing alcohol and identifying hazardous alcohol use in patients with hypertension managed in PHC. The GPs advocated adopting PEth as a routine test in the treatment of hypertension. However, PEth needs to be used with care to maximise benefit and minimise harm.

## How this fits in

It is common to use the alcohol biomarker phosphatidylethanol (PEth) to identify hazardous alcohol use in Swedish PHC. This study explored GPs’ experiences of using PEth routinely in hypertension management. GPs found that PEth changed their routines for identifying hazardous alcohol use, improved the dialogue about alcohol, and made it easier to assess whether alcohol was contributing to the patient’s hypertension. Patients seldom reacted negatively to PEth, but since it could be experienced as stigmatising, PEth should be used with care. GPs in the study advocated routine use of PEth in primary care, but ethical principles should guide its use.

## Introduction

High systolic blood pressure is the most important contributor to the global burden of disease, and alcohol the seventh.^1^ Proper hypertension treatment is crucial in preventing various cardiovascular conditions and dementia.^2^ Most patients with hypertension are treated in PHC,^3^ but only 37% reach their target blood pressure.^4^ Hazardous alcohol use increases the risk for adverse health consequences,^5–7^ but GPs fail to recognise 60–70% of patients who have such use.^8^ Regular alcohol consumption has a causal and dose-dependent impact on blood pressure,^9,10^ with 5–24% of hypertension cases attributed to alcohol use.^11^

Hypertension guidelines include awareness of harmful lifestyles,^2^ but barriers exist in enquiring about alcohol use including the following: stigmatisation;^12–16^ time constraints;^17,18^ and patients’ under-reporting of alcohol use.^17^ The Alcohol Use Disorders Identification Test (AUDIT)^5^ is a widely-used,^19^ validated screening questionnaire for identifying hazardous and harmful use but studies have identified barriers for implementation in PHC,^20^ and suggest low utilisation rates.^21^ Both AUDIT and PEth are recommended in Swedish health care.^22,23^

PEth, a direct alcohol biomarker,^24^ detects alcohol use^25,26^ and chronic heavy consumption^27,28^ with high sensitivity and specificity, and is suitable for monitoring abstinence.^25^ Conclusive evidence is lacking about whether PEth can identify binge drinking.^28^ Studies from different countries have demonstrated the usefulness of PEth in various clinical situations,^29,30^ and the use of PEth is increasing, even if it is difficult to obtain an overview of exactly where it is used. PEth does not appear to be used routinely in any clinical practice.^31^ However, in Swedish health care PEth is used for conditions such as liver diseases, hypertension, and mental illness.

GPs receive PEth test results in their routine laboratory test list, providing the PEth value and its categorisation (low, moderate, or excessive). Interpretation involves evaluating quantity, frequency, and timing of alcohol consumption.^24^ Swedish PHCCs pay 24 EUR (approximately 20.60 GBP) per PEth test, making them 27–60 times costlier than plasma glucose and creatinine tests.

The use of alcohol biomarkers in clinical situations^32^ should be guided by ethical principles.^33^ Swedish laws^34,35^ emphasise respecting patients' autonomy and involving them in the decision-making process.

To the best of the authors' knowledge, there are no studies on whether GPs find PEth a useful tool for identifying hazardous alcohol use in connection with hypertension. The present study aimed to explore GPs’ experiences of using PEth in the context of clinical hypertension management in PHC.

## Method

### Study design

In this qualitative study, GPs recruited from Swedish PHCCs participated in digital focus group interviews^36,37^ in 2021. Findings are reported in accordance with the Standards for Reporting Qualitative Research.^38^

### Sampling strategy

GPs and residents in general medicine (hereafter GPs) (*n* = 15) were recruited via social media groups and advertisements in Swedish journals for GPs, the researchers’ network, and personal contacts. The sample was purposive: experiences of using PEth routinely at annual hypertension check-ups or often (implemented PEth but not routinely). This was checked verbally during recruitment. One GP dropped out because of an urgent incident, and two because of scheduling conflicts. Therefore, there was a sample size of *n* = 12.

### Study participants

GPs working at PHCCs located in urban, suburban, and rural areas were represented. A description of participants is presented in Table 1.

**Table 1.** Participant characteristics, self-reported

Participant	Age, years	Sex	Year of medical certificate	Years (*n*) as specialist in general medicine	Years (*n*) active in PHC	Other specialist competence	Further education alcohol
**A**	35	M	2013	3	7	No	Half day
**B**	45	F	2009	3	12	No	More than half day
**C**	45	F	2011	4	10	No	No
**D**	47	M	2003	10	16	No	More than half day
**E**	59	M	2001	13	20	No	More than half day
**F**	43	F	2012	0	3	No	No
**G**	75	M	1973	42	48	No	More than half day
**H**	47	F	2002	11	16	No	Half day
**I**	36	F	2019	0	2	No	No
**J**	38	F	2012	2	9	No	No
**K**	31	M	2018	0	3.5	No	More than half day
**L**	43	M	2006	10	15	No	More than half day

### Ethical considerations

All relevant study information was presented to the GPs orally and in writing. GPs provided written informed consent before the focus group interviews. Data were managed to protect GPs’ privacy.

### Data collection and processing

Twelve GPs from 10 different PHCCs in Sweden participated in five focus group interviews. The interviews lasted between 60 and 90 minutes and had two to three participants. A questioning route^37^ was developed specifically for the study. The first author (ÅT) wrote the first draft and revised it together with four of the other authors (MH, JA, JH, and LL). It was again revised after testing on a separate group of GPs and after input from one additional author (CS). Topics and interview questions are presented in Table 2 and the entire questioning route in Appendix 1 (see Supplementary File).

**Table 2.** Topics and interview questions in the questioning route

1. How the GP addresses alcohol in the meeting with the patient
2. Experiences of using PEth as a part of the annual check-up and treatment of hypertension. Pros and cons? What was the rationale for starting to use PEth? Have you received education about PEth? How is it to interpret PEth? Does the use of PEth alter the number of patients you detect with hazardous alcohol use and the number of patients who receive further management?
3. How the dialogue is influenced when PEth is used. What happens when you communicate the test result to the patient? How does the patient react and how are they informed? What questions does the patient ask? Does the patient refuse to have PEth analysed?
4. Experiences and thoughts about PEth as a routine test. Pros and cons? Does the possibility of detecting hazardous use increase or decrease with PEth as a routine test?
5. Experiences of other methods to detect hazardous alcohol use compared with PEth?
6. What factors affect whether you offer support to the patient to reduce alcohol consumption?

PEth = phosphatidylethanol.

ÅT moderated the focus groups on the digital platform Teams M365 (version 1.5.00.21463). CS was an observer and encouraged dialogue. The interviews continued until they did not add any new information, at which point data saturation^39^ had been reached. Two copies of the recorded interviews were saved (Teams and audio-recorder). An external writing company transcribed four interviews verbatim and ÅT one, using Microsoft Office 365 Word software.

### Analysis

Data were analysed with inductive qualitative content analysis,^40^ a part of the hermeneutic paradigm.^41^ All interview transcripts were merged into one Microsoft Office 365 Excel spreadsheet. Text relevant to the aim of the study was divided into meaning units,^40^ which were distilled into condensed text.^40^ The condensed text was abstracted and labelled with a code^40^ reflecting the manifest content. Codes reflecting the same concept were grouped into categories. Categories describing the same phenomenon were abstracted and grouped into sub-themes. Finally, the authors developed, through interpretation, an overarching descriptive theme reflecting the latent content of the text.^40^ The sub-themes and theme were named by the authors.

During the study, ÅT took notes about key findings, reflections, and theoretical reasoning and then used them iteratively during the analysis and writing to search for patterns and to check whether proposed categories and sub-themes fit the data. ÅT (the main person responsible for coding) performed the analysis in collaboration with LL and CS. All authors participated in the entire research process. The analytical iterative process^40^ is illustrated in Table 3.

**Table 3.** Example of the analytical process with meaning unit, code, category, and sub-theme

Meaning units	Codes	Category	Sub-theme
*'… sometimes surprised that yes, the prejudices that you had about people and then you see that they have high PEth, I didn't expect that.'*	Amazement at high value	Often surprised	Serving as an eye-opener
*'… this affects your health and that, or there is a risk that it affects your health, and then you get an entrance into the conversation that you might not otherwise have gotten.'*	Entrance you might have missed	Getting straight to the point	Improving the dialogue
*'… quite a lot of extra work to sort of collect consent, and then maybe you really, so theoretically you should maybe have consent for everything you do.'*	Consent for other things	Feeling ambivalent about informing patients and ask for permission	Using with care
*'There is the occasional time I have come across that patients have questioned why I have taken, taken PEth.'*	Occasionally questioned why	Sorting out the rare negative reactions	Learning by doing

PEth = phosphatidylethanol.

### Research team and reflexivity

ÅT, who is a PhD student and GP, is trained in Motivational Interviewing and has experience of working with lifestyle habits in a variety of professional contexts. LL and CS are district nurses with experience in clinical practice and in health promotion research. The professional experiences of the research team have affected how the study was performed and interpreted. ÅT has limited experience of using PEth in clinical work, and CS and LL have no experience of using the test. They could therefore be open-minded about GPs’ experiences of using PEth. During the interviews and analysis, the authors were influenced by the GPs' favourable views on utilising PEth, so they actively strove to maintain objectivity in assessing their experiences.

### Trustworthiness

To enhance credibility, the participants were encouraged to illustrate their statements with practical examples. ÅT listened to all recordings and read the transcripts multiple times. CS and LL also read all the transcripts. CS, the observer, played an active part in the analysis, which enhanced credibility. The codes, categories, and sub-themes were continuously discussed and revised during analysis and writing. Quotes from different GPs increased trustworthiness. The authors included GPs, a medical doctor and public health expert, a physiotherapist, and district nurses, all with clinical PHC experience and experience conducting research in PHC.

## Results

The main theme represents descriptive latent content, and sub-themes and categories reflect the manifest content in the data.^40^ Quotations illustrate the theme and categories. Capital letters in parentheses at the end of the quotation indicate the speaker. The theme, sub-themes, and categories are presented in Table 4.

**Table 4.** Categories, sub-themes, and theme describing GPs’ experiences of using PEth when managing hypertension

Category	Sub-themes	Theme
Often surprised	Serving as an eye-opener	I don’t hesitate anymore
Helping GPs obtain a reliable picture		
Making it easier to treat correctly		
Getting straight to the point	Improving the dialogue	
Avoiding the difficulty of quantifying		
Facilitating individualisation		
Feeling ambivalent about informing patients and asking for permission	Using with care	
Risk of increasing stigmatisation		
Needing to de-dramatise		
Deciding when to order PEth	Learning by doing	
Sorting out the rare negative reactions		

PEth = phosphatidylethanol.

### Theme: I don’t hesitate anymore

PEth has changed routines for identifying hazardous use, and the GPs no longer hesitated to use the test. The theme 'I don’t hesitate anymore' reflected the disappearance of the fear that PEth might upset the patient as this rarely occurred, and the positive effects of PEth predominated. PEth helped the GPs identify hazardous use and improved the dialogue about alcohol. However, PEth needs to be used with care to avoid negative feelings in patients:

*'I'm not embarrassed about it* [PEth] *anymore, partly because I'm a bit more used to using it, partly because I see that it does, it gives me a lot of information, and it can do a lot of good. And that I, this fear that people will actually get angry has mostly disappeared over time as well, because they seldom do. When you bring it up in the right way.'* (J)

The theme is underpinned by the following four sub-themes: serving as an eye-opener; improving the dialogue; using with care; and learning by doing. 'Serving as an eye-opener' and 'improving the dialogue' reflected the positive effects of PEth, motivating the GPs to use the test more often. 'Using with care' captured how the GPs handle PEth to not harm the patients. GPs' difficulties in assessing which patients had hazardous alcohol use and that patients seldom reacted negatively to PEth, is reflected in 'learning by doing'.

### Sub-theme: Serving as an eye-opener

PEth made it easier to identify patients with hazardous alcohol use and therefore helped the GPs manage hypertension correctly.

#### Often surprised

The PEth result could surprise the GPs, as it often did not correspond to what they had expected. They had the preconception that they could easily judge whether patients had hazardous alcohol use based on clinical experience, but the PEth results often showed that they had misjudged consumption:

*'*[You] *had these who you didn't think about, and then suddenly when you've had them for a few years, oh no, I haven't checked PEth, and then you do the test, and then it’s elevated.'* (E)

GPs described the PEth result as a wakeup call making many patients reflect on their alcohol consumption:

*'"I probably drink a glass of wine every now and then,”* [says the patient]. *And then when you see a very high PEth, and then just, “How much is it?” And then they start to think, “Yes, yeah, yeah, but it’s almost every day, and a bag in box disappears in a week”.'* (K)

#### Helping GPs obtain a reliable picture

PEth helped the GPs obtain a reliable picture of whether alcohol might be contributing to a patient’s hypertension. They wanted to believe the patients’ accounts of their alcohol use but found that patients sometimes had difficulty quantifying the amount they drank, reported incorrectly because of memory loss or social desirability, or denied overconsumption. AUDIT was more time-consuming and a blunter tool than PEth, and seldom came to use.

#### Making it easier to treat correctly

Because PEth gave a more reliable picture of the alcohol consumption, the test results also made it easier to treat hypertension and other diseases correctly:

*'We treat hypertension but in fact it’s alcohol abuse we should be treating, so we’re doing very wrong for many years to come.'* (J)

Although PEth had some disadvantages, it provided a valuable contribution to the medical assessment. GPs realised that PEth tests are relatively expensive but they perceived PEth as economically advantageous because it promoted patients’ health in the long term.

### Sub-theme: Improving the dialogue

PEth was time efficient and facilitated individualised, high quality dialogue with patients.

#### Getting straight to the point

When the GPs used PEth, they could directly focus on the most relevant issue. PEth helped start the patient–doctor dialogue regardless of whether the result reached the limit of hazardous use or not. And even when the patient declined a PEth test a dialogue could start:

*'Why* [isn’t the patient willing to take a PEth test]*? "Yes, I know it’ll be high," she might say, and anyway, it’ll start a conversation.'* (D)

#### Avoiding the difficulty of quantifying

By using PEth, GPs avoided the difficulty of quantifying the alcohol consumption. According to the GPs, patients’ answers could be imprecise or even meaningless owing to difficulties of quantifying alcohol consumption, GPs therefore questioned the usefulness of a medical history they could not trust.

#### Facilitating individualisation

PEth facilitated individualisation because the GPs interpreted the PEth result as an indicator of how the patient’s body had reacted to their alcohol consumption and then adapted the dialogue to the individual patient:

*'You wouldn't know, you can always say that, well, this is how it is with alcohol and blood pressure, but now* [with PEth] *you can say that we see that this . . . applies to you.'* (A)

### Sub-theme: Using with care

PEth may increase stigmatisation, so the GPs strove to use and talk about PEth with care.

#### Feeling ambivalent about informing patients and asking for permission

The GPs in the study were aware that healthcare professionals are required to provide care in consultation with patients and to respect patients’ autonomy. However, guidelines do not clarify when and how to ask about tests in general or PEth in particular. They routinely informed patients that hypertension treatment includes medically relevant blood tests but could feel ambivalent about specifically informing patients about PEth and asking their permission to order it. The consent for testing was given orally.

They found it important to be transparent but also stated that they did not always inform patients about other laboratory tests. Sometimes GPs informed patients about PEth tests and sometimes they did not, depending on the individual situation. If they had already indicated that alcohol was important by bringing it up at the visit, or if PEth was a routine test, then they did not feel it was mandatory to specifically inform the patient about PEth and ask for permission to order it. They could even feel that asking for permission unnecessarily dramatised the test.

GPs reported that originally, they typically asked before ordering PEth. With time, they found that patients were seldom upset if they forgot to ask for permission before ordering PEth and seldom upset about the test at all. This experience could lead GPs to ask for permission less frequently or to stop asking for permission. They seldom perceived it as a problem to use PEth routinely in connection with hypertension, without specifically asking for permission about PEth:

*'I’ve started to just simply take it without asking. Haven’t perceived that there’s been any major problem, either.'* (K)

#### Risk of increasing stigmatisation

A PEth result could disclose patients’ actual alcohol consumption, which could feel stigmatising. GPs were therefore careful to explain that the purpose of the test was to help, not to judge, the patient. But no matter how carefully the GPs handled PEth, it could still feel sensitive:

*'You can sometimes sit with test results that feel a little extra sensitive, and right now when we're talking about PEth, it can be* [such a result]*.'* (E)

GPs could fear that they had shown mistrust in the patient by ordering the test at all, instead of only talking about alcohol. Patients have also felt singled out when PEth was ordered just for them, feeling that they were suspected of drinking too much. Treating PEth as a test requiring special permission could increase stigmatisation because it implied that alcohol is such a sensitive topic that the doctor had to tiptoe around it. This could be counterproductive to an open dialogue.

#### Needing to de-dramatise

GPs strove to de-dramatise PEth, they communicated about the test and the negative effects of alcohol in a respectful, non-judgmental, non-moralising way that was accurate and easy to understand. They explained PEth as the following: an alcohol test, a test related to blood pressure, a liver test, or a test that would help them treat the patient’s hypertension correctly. If the patient’s PEth value was elevated, instead of saying, *'You drink too much,'* they might explain that:

*'We see that your body feels the amount that you drink'* (A).

Even if the explanation about the body was not totally correct, they found it useful. The most powerful way to de-dramatise PEth results and decrease the risk of stigmatisation was to use it routinely.

GPs also de-dramatised the situation when what a patient reported about their drinking did not correlate with a later PEth result. They expected the patient to feel ashamed, angry, or upset. GPs de-dramatised the situation by leaving the previous report behind, showing empathy, and avoiding an argument about the real alcohol consumption. GPs used Motivational Interviewing skills to de-dramatise PEth and talk about alcohol without moralising or accusing.

### Sub-theme: Learning by doing

By using PEth, the GPs had learnt that negative reactions were rare and now advocated its routine use.

#### Deciding when to order PEth

The GPs found it hard to decide which patient they should order PEth for. They based the decision on previous knowledge of the patient, medical problems associated with alcohol, and prejudice. They seldom expected that women, older patients, or patients who they knew well would have hazardous alcohol use:

*'No, I’ve totally stopped trying to judge, judge patients in advance, who I should order it for and not, it kind of doesn’t work.*' (C)

Previously, GPs ordered PEth when they strongly suspected overconsumption; therefore, they often missed hazardous use. Now they used PEth more liberally and advocated its routine use, as they were comfortable with using it, thought it added valuable information, and seldom upset patients.

#### Sorting out the rare negative reactions

Almost all GPs had occasionally treated patients who questioned the PEth result or became upset or offended. However, the negative reactions were considerably rarer than the GPs had anticipated:

*'When I talk with my colleagues about it ... if I can get together one, two, three people who’ve been, gotten irritated about this, then it’s not more in, in* [inaudible] *several hundred tests, I would say.*' (G)

PEth could adversely affect the patient–doctor relationship during the visit, but according to GPs, not permanently damage the relationship. If the GPs handled it well and talked it through, the dialogue could move on, and the disturbance in the relationship was sorted out. The patient continued to visit the GP, and sometimes even expressed thanks:

*'So, it’s never a problem when they* [those who get upset] *leave the room, then we've already solved it, and we're over it and have moved on.*' (B)

## Discussion

### Summary

PEth is growing in popularity in Swedish PHC and in other countries, but there is scarce knowledge about why this is the case. This study has increased the understanding of how GPs experience using PEth in the management of hypertension and thus added novel knowledge to the literature. The study has also illuminated a lack of guidance for GPs in Sweden about how to use alcohol biomarkers that reveal sensitive information.

The main findings of the study showed that the positive effects of PEth in routine hypertension management outweighed the negative effects. Moreover, PEth is an eye-opener that improves the dialogue about alcohol. It seldom upsets, but could increase stigmatisation and therefore should be used with care guided by ethical principles. However, negative reactions from the patients were rare. GPs no longer hesitated to use PEth and advocated the routine use of PEth in connection with hypertension.

### Strengths and limitations

Focus groups stimulate interaction between participants and can provide rich data.^36,37^ As the focus group interviews were performed digitally, data collection from different parts of Sweden was facilitated. Some of the focus groups were small. Larger groups might have stimulated more vigorous discussions of varied experiences and perspectives. In the assessment, however, the information power was high.^42^ To increase credibility and transferability, the study aimed to recruit GPs with both positive and negative experiences. Despite these efforts, participating GPs did not spontaneously describe highly negative experiences of PEth. A possible disadvantage of focus groups is that negative and sensitive information may not be revealed, though in the assessment GPs were unlikely to feel that it was sensitive or negative to talk about the topic of the study. Through follow-up questions, the researchers were also able to initiate discussions about adverse experiences of the test and the open and supportive group dynamics allowed participants to bring up negative aspects. There is, however, a risk that focus groups will obscure individual experiences.^43^ The GPs in the focus groups were men and women of a variety of ages. They differed in the length of time they had worked in PHC, the regions of Sweden where they lived and worked, and the length of their education about alcohol. Diverse author backgrounds fostered varied perspectives during data analysis. To increase credibility, ÅT was engaged in the entire study process.

The study has provided knowledge about what GPs believe patients think. However, it has not illuminated patients’ actual perspectives. Therefore, the authors are currently conducting a study about patients' experiences of PEth.

### Comparison with existing literature

#### Patient–doctor relationship

Fear of disturbing the patient–doctor relationship can make GPs reluctant to address alcohol use.^44–47^ The finding that GPs think PEth seldom obstructs the patient–doctor relationship is therefore important. The public largely agrees that healthcare professionals should routinely discuss alcohol with patients, but people with moderate or hazardous alcohol use are less positive.^48^ One might therefore expect that PEth would upset patients, but the results show that negative reactions were rare.

The study has described features of PEth that improved the dialogue about alcohol and motivated patients to change alcohol habits. Individualised advice is motivating^49^ compared with general advice, and PEth facilitated an individualised dialogue and helped patients find their own reasons for change. Such intrinsic motivation is a prerequisite for change^50^ according to Motivational Interviewing.

Motivational Interviewing^51^ can increase *'client change talk*'^50^ and help patients reduce alcohol consumption.^52,53^ When the GPs talked about alcohol based on PEth, they employed a variety of Motivational Interviewing components^54^ including the following: cooperating with the patient; respecting the patient’s autonomy; showing empathy; avoiding dissonance; asking for permission to raise the subject; using open questions; exploring the patients’ knowledge; and adjusting the information they provided on the basis of that knowledge.

#### Stigmatisation

The study results are coherent with previous knowledge of the stigma^13,14,55^ that can be associated with overconsuming alcohol.^55^ Concerns about stigmatising patients can be a barrier to implementing screening and brief interventions for alcohol overconsumption in PHC.^16,56^ Fear of being stigmatised can inhibit treatment seeking.^44^ A clinically relevant finding of the study is that GPs think the routine use of PEth, in check-ups for patients with hypertension, could decrease the stigma about testing for alcohol overconsumption.

GPs in the study strove to adhere to the ethical principle of nonmaleficence.^33^ They emphasised that it was crucial to be able to talk about PEth and alcohol in a respectful way, explained PEth in an easy way, and pointed out the medical indication.

#### Alcohol biomarkers and ethical principles

Ethical considerations (maximise benefit and minimise harm)^33^ are a part of GPs’ everyday clinical practice. The finding that PEth made it easier to follow guidelines for hypertension management indicated that PEth can help GPs maximise benefits for patients.

In Sweden, healthcare professionals provide care in consultation with patients, whose autonomy must be respected.^34,35^ Patients may not be subjected to medical procedures without their consent;^35^ this can include blood tests^57^ such as PEth. Patients must be aware of which test is ordered, medical indications for the test, and they have the right to decline them.^35^ Additionally, patients should be informed about the purpose of screening instruments such as AUDIT.^20,58^ However, guidelines do not always clarify how GPs should implement these laws and principles in routine practice. The GPs in the study often asked each patient for consent before ordering a PEth test, but there were occasions when they did not. If PEth was a routine test at hypertension check-ups, they might not ask for consent. The same was true if they had previously talked about alcohol with the patient. Since, in their experience, patients were seldom upset about PEth, they might not always ask. GPs could even think that it was more respectful not to ask for consent, as asking might make patients feel singled out.

Negative reactions to PEth without asking for permission were few, easy to resolve, and the patients continued to visit the GPs. Nevertheless, according to laws, patients need to consent before medical procedures. The study has thus illuminated a lack of routines for obtaining consent in ordinary care.

#### PEth and AUDIT

Many countries recommend AUDIT, which is used in a variety of settings.^19,58^ PEth is also used in clinical contexts in many countries,^29^ but the degree to which it is implemented varies. Using both PEth and AUDIT might help increase detection of alcohol use in patients,^59^ and instruments, such as AUDIT, and biomarkers, such as PEth, can play a role in verifying the diagnosis of alcohol dependence.^60^ However, it appeared that neither PEth nor AUDIT is recommended or used as a routine component of hypertension check-ups.

GPs miss a large proportion of hazardous alcohol use.^8^ Questionnaires are a validated option, but GPs seem to seldom choose to use them.^15,21,47^ This could be for a variety of reasons including time constrains, questionnaires are challenging to integrate into daily routines,^20^ and awareness that patients under-report alcohol use in questionnaires.^17,47^ Problems with under-reporting alcohol consumption are an important reason for the increasing interest in finding and using direct alcohol biomarkers to obtain objective measurements of alcohol use^28^ and such biomarkers can confirm or disconfirm the self-reported alcohol consumption.^61^

Direct alcohol biomarkers are preferred over indirect as they give a more correct assessment of consumption.^32^ Additionally, PEth has almost complete specificity and higher sensitivity than unspecific biomarkers and the indirect carbohydrate-deficient transferrin.^27,62^ A limitation of PEth is the lack of international consensus on cut-offs^24^ but interpretation guidelines for PEth exist.^24,63^

For the GPs in the current study, PEth seemed to have filled the need for a practical tool for identifying hazardous alcohol use in PHC patients. It provided a reliable picture of patients’ alcohol consumption. PEth improved efficiency because it helped GPs get directly to the point and individualised the dialogue. However, PEth should be used with care, guided by ethical principles. PEth has changed GPs' routines to identify hazardous alcohol use and they have advocated its routine use.

### Implications for research and practice

This study has added novel knowledge about using PEth in routine hypertension care and may initiate discussions about implementing PEth in the treatment of other lifestyle-related disorders in PHC. Guidelines state that lifestyle factors should be addressed in the management of hypertension but are often vague about how to address it. This study has suggested that PEth may be a tool for bridging the gap between what needs to be done and how to do it.

However, the study illuminated a need to clarify guidelines to ensure that ethical principles and laws are followed when using PEth. Also, studies of patients’ own perspectives on PEth are crucial. The findings have suggested that PEth provides a valuable contribution to hypertension check-ups if used with care. Future research should continue to investigate whether and how to use PEth in PHC.

## Data

The data that support the findings of this study are available on reasonable request from the corresponding author. The data are not publicly available due to privacy or ethical restrictions.
